# Research on Biodiversity and Climate Change at a Distance: Collaboration Networks between Europe and Latin America and the Caribbean

**DOI:** 10.1371/journal.pone.0157441

**Published:** 2016-06-15

**Authors:** Olivier Dangles, Jean Loirat, Claire Freour, Sandrine Serre, Jean Vacher, Xavier Le Roux

**Affiliations:** 1 Institut de Recherche pour le Développement (IRD), UR 072, EGCE, UPR 9034, CNRS 91198 Gif-sur-Yvette Cedex, France and Université Paris-Sud 11, 91405, Orsay Cedex, France; 2 Pontificia Universidad Católica del Ecuador, Facultad de Ciencias Exactas y Naturales, Av. 12 de Octubre y Roca, Quito, Ecuador; 3 Institut de Recherche pour le Développement (IRD), 44 bld de Dunkerque, 13572, Marseille, France; 4 Institut de Recherche pour le Développement (IRD), UMR PALOC, MNHN, Paris, 75005, France; 5 Microbial Ecology Centre, INRA (UMR 1418), CNRS (UMR 5557), Université de Lyon, Université Lyon1, 43 bd du 11 nov 1918, Villeurbanne, France; 6 BiodivERsA, Fondation pour la Recherche sur la Biodiversité (FRB), 195 rue Saint Jacques, 75005, Paris, France; University of Colorado, UNITED STATES

## Abstract

Biodiversity loss and climate change are both globally significant issues that must be addressed through collaboration across countries and disciplines. With the December 2015 COP21 climate conference in Paris and the recent creation of the Intergovernmental Platform on Biodiversity and Ecosystem Services (IPBES), it has become critical to evaluate the capacity for global research networks to develop at the interface between biodiversity and climate change. In the context of the European Union (EU) strategy to stand as a world leader in tackling global challenges, the European Commission has promoted ties between the EU and Latin America and the Caribbean (LAC) in science, technology and innovation. However, it is not clear how these significant interactions impact scientific cooperation at the interface of biodiversity and climate change. We looked at research collaborations between two major regions—the European Research Area (ERA) and LAC—that addressed both biodiversity and climate change. We analysed the temporal evolution of these collaborations, whether they were led by ERA or LAC teams, and which research domains they covered. We surveyed publications listed on the Web of Science that were authored by researchers from both the ERA and LAC and that were published between 2003 and 2013. We also run similar analyses on other topics and other continents to provide baseline comparisons. Our results revealed a steady increase in scientific co-authorships between ERA and LAC countries as a result of the increasingly complex web of relationships that has been weaved among scientists from the two regions. The ERA-LAC co-authorship increase for biodiversity and climate change was higher than those reported for other topics and for collaboration with other continents. We also found strong differences in international collaboration patterns within the LAC: co-publications were fewest from researchers in low- and lower-middle-income countries and most prevalent from researchers in emerging countries like Mexico and Brazil. Overall, interdisciplinary publications represented 25.8% of all publications at the interface of biodiversity and climate change in the ERA-LAC network. Further scientific collaborations should be promoted 1) to prevent less developed countries from being isolated from the global cooperation network, 2) to ensure that scientists from these countries are trained to lead visible and recognized biodiversity and climate change research, and 3) to develop common study models that better integrate multiple scientific disciplines and better support decision-making.

## Introduction

There is an increasing need to address global issues like biodiversity loss and climate change [1]. However, both issues are particularly complex, and addressing them requires skills and research inputs that span countries and disciplines [2]. Interdisciplinary teams have largely been promoted by funding programmes that direct research to development and politically desired goals [3]. Though collaborative research teams are common in studies of global, multi-faceted problems, international interdisciplinary research networks are not easy to build [4]. In particular, creating an effective international, interdisciplinary team is not as simple as bringing together a group of scientists from different disciplines and countries. It requires the development of common research languages, methods and models across both team members and countries [5].

Europe is the major donor for research and development cooperation in Latin America and the Caribbean (LAC), is the largest foreign direct investor in LAC, and is a key promoter of regional integration [6]. In the context of the EU strategy to stand as a world leader in tackling global challenges, the European Commission (EC) has indeed strongly promoted ties between the EU and LAC in science, technology and innovation. More than 750 LAC-based researchers have been funded through collaborative projects with European partners for a total €100 million through the 7th Framework Program (FP7) for research and innovation (2007–2013, www.eeas.europa.eu/lac; see [7] for more details). As an example, CLARIS-LPB project (2008–2012 http://www.claris-eu.org/) brought 9 partners from LAC and 11 from EU together, to build possible scenarios on climate change on La Plata Basin and design adaptation strategies. The main areas of common interest promoted by the FP7 were renewable energies, climate services, bio-economy, marine research, ICT and health. Following the adoption by the EU-LAC Madrid Summit of the Joint Initiative for Research and Innovation (JIRI) in 2010, the EC has intensified its support for policy dialogue on science and technology. In particular, this led to the launch of the ALCUE-Net project (2013-2017- www.alcuenet.eu) with the main objective to establish a bi-regional platform between actors involved in research and innovation, relevant stakeholders from the public and private sectors, and civil society so that a long-standing EU-LAC dialogue on science and technology could be implemented. The EC also supported the ERANet-LAC (http://eranet-lac.eu), which is a network of the EU and LAC Countries strengthening the bi-regional partnership in science, technology and innovation by planning and implementing concrete joint activities such as joint calls for research projects.

Beyond European programmes and initiatives, collaborative research between the ERA and LAC at the interface of biodiversity loss and climate change is also spurred by bilateral schemes that promote research between one European country and one LAC country. For instance, Brazil and Mexico were among the 14 countries for which the French National Research Agency (ANR) dedicated a bilateral scheme in 2014. For several years, the German Research Foundation (DFG) has operated a joint funding program with the Brazilian partner organisations FAPESP in the state of São Paulo and FAPEMIG in the state of Minas Gerais based on bilateral cooperation agreements.

However, it is not clear how these European, trans-continental and bilateral research programmes impact scientific cooperation at the interface of biodiversity and climate change. Political leaders in the European Union (EU) and in LAC have long recognised the importance of incorporating scientific knowledge into proposed solutions to the increasingly complex challenges that biodiversity loss and climate change pose to society at both local and global scales [8, 9]. More knowledge-based solutions to these challenges are needed with the recent creation of the Intergovernmental Platform on Biodiversity and Ecosystem Services (IPBES), the debates associated with the December 2015 Climate Change Conference in Paris (COP21), and the need for the European Research Area (ERA) to explore nature-based solutions [10] and climate services [11]. ERA and LAC countries together host a great proportion of the world’s biodiversity [12] and are expected to experience significantly different and less predictable climates over time [13]. Therefore, cooperation at the interface of biodiversity and climate change should interest both, and demand holistic and regional approaches. But the outcome of the different research programmes developed for promoting ERA-LAC research collaboration remains to be evaluated, in particular for hot topics like the interactions between biodiversity and climate change.

Bibliographic analyses are useful for quantifying bi-regional cooperation in scientific research [14, 15]. In particular, as scientific publications are the product of collaboration among researchers and institutions, they can provide an overview on the structure and dynamics of research networks. Therefore, the results of efforts to promote international research collaboration can be assessed by analysing networks of co-authors and characteristics of collaborations, such as temporal trends, and the geographic areas and research domains that they cover. Here, we analysed publications involving authors from the ERA and LAC published between 2003 and 2013 to study bi-regional research collaboration at the interface between biodiversity and climate change. We evaluated the relative importance of ERA-LAC collaboration compared to collaborations with other regions, the temporal evolution of the importance of ERA-LAC collaboration, the nationality of corresponding authors, and the type of research domain covered. We discuss the implications of our study to guide further development of ERA-LAC research collaborations in the future.

## Materials and Methods

### Bibliographic review

Using the Web of Science version of the Thomson Reuters citation databases (WoS, webofknowledge.com), we searched the peer-reviewed literature (articles and reviews) published between 2003 and 2013 that focused on biodiversity, climate change and both together, and identified papers originating from ERA and LAC countries. The Web of Science platform draws from several online databases, three of which were particularly relevant for our search: two from the core collection (Science Citation Index, SCI: 7,100 journals; Social Science Citation Index, SSCI: 2,100 journals, etc.) and one from the Arts & Humanities Citation Index (AHCI: 1,700 journals). We found only four additional references by using other databases, including SciELO (mainly for South America), Scopus, Social Science Research Network, BioOne and MUSE.

We retrieved all publications with at least one author from a country within LAC (33 countries: Antigua and Barbuda, Argentina, Bahamas, Barbados, Belize, Bolivia, Brazil, Chile, Colombia, Costa Rica, Cuba, Dominica, Dominican Republic, Ecuador, El Salvador, Grenada, Guatemala, Guyana, Jamaica, Haiti, Honduras, Mexico, Nicaragua, Panama, Paraguay, Peru, Saint Kitts and Nevis, Saint Lucia, Saint Vincent and the Grenadines, Suriname, Trinidad and Tobago, Uruguay, Venezuela) and all publications with at least one author from a country within the ERA (28 countries from the EU plus 14 associate members: Austria, Belgium, Bulgaria, Croatia, Czech Republic, Cyprus, Denmark, Estonia, Finland, France, Germany, Greece, Hungary, Ireland, Italy, Latvia, Lithuania, Luxembourg, Malta, the Netherlands, Portugal, Poland, Romania, Slovakia, Slovenia, Spain, Sweden, United Kingdom, plus Albania, Bosnia, Faroe Islands, Israel, Iceland, Liechtenstein, Macedonia, Montenegro, Moldova, Norway, Serbia, Switzerland, Turkey). The publications with at least one author from a LAC country and at least one author from an ERA country were considered an ERA-LAC co-publication, as were publications authored by at least one scientist who was affiliated with organizations in both the ERA and LAC. To obtain baseline comparisons, we also performed a search for other continents, i.e. North America, Africa, Asia and Oceania.

As biodiversity is a broad, multi-faceted concept, we used the following keyword profile to identify biodiversity-related publications: TOPIC = (biodivers*, biological diversity, species richness, species diversity, taxonom*, phylogen*, animal diversity, mammal diversity, bird diversity, fish diversity, reptile diversity, amphibian diversity, frog diversity, insect diversity, plant diversity, weed diversity, microbial diversity, bacteria* diversity, fung* diversity, virus diversity, ecosystem diversity, habitat diversity, landscape diversity, biological conservation, species conservation, habitat conservation, genetic resource*, functional diversity, functional trait*, invasive species, biological invasion*, functional type*, functional group*). To search for publications on climate change, we used the following keywords: TOPIC = (climat* chang*, global warming, climat* variab*, climat* warming, extreme event*). We cross-referenced the two searches to identify publications at the interface of biodiversity and climate change and checked that all articles were related to these two specific topics. To obtain baseline comparisons, we also performed a search for other topics, i.e. all the publications not using any of the above-mentioned keywords.

All records were imported into an Excel dynamic database. Each record was tagged with its corresponding WoS section, SCI, SSCI or AHCI, a repeatable attribute as a relatively high level of record duplication exists between the three sections. The database was cleaned to avoid duplications and remove errors and inconsistencies (for example, in the country name, in the affiliations and address fields, see S1 Table).

### Data analysis

#### Co-publication network analysis

We used the author addresses listed in the papers to determine the international networks of researchers. For each paper, we recorded information on all authors and identified the collaborating countries. Finally, we computed a triangular matrix to identify the strength of the links between each pair of countries based on the number of papers co-authored by these countries. Co-publication network mapping and analysis were performed using the open source Gephi software ([16], http://gephi.org).

#### Mapping and co-authoring network analysis

We performed two-dimensional spatial mapping of ERA-LAC co-authorship networks working at the interface of biodiversity and climate change using the Force-Atlas 2 algorithm in Gelphi. This algorithm creates a visual representation of nodes (countries) connected by edges (based on co-authorship) according to the following rules: 1) node size represents the number of publications, 2) all nodes are attracted to the center (the country with the highest number of publications), 3) all nodes repel each other (to prevent visual overlapping of the nodes), 4) all nodes that are connected by an edge attract each other, according to the weight of the edge (the number of publications with co-authorship between the two countries/regions). Two nodes are thus spatially closer if there are many collaborative publications. To examine the dynamics of the development of ERA-LAC co-authorship networks that were working at the interface of biodiversity and climate change over the last 11 years, we built maps for two different periods: 2003–2008 and 2008–2013.

We also created two-dimensional maps of the co-publication networks at the global level, looking at collaborations between the ERA, LAC, other European countries, North America, Africa, Asia, Oceania. As these networks were drawn on a world map background, only node size and edge weight were represented.

We computed two indicators to evaluate the role of each country within the ERA-LAC co-publication network [5]: (i) the scientific production level, i.e. the number of publications produced, normalized by the maximum number observed, and (ii) the betweenness centrality, normalized by the maximum value observed, which indicates how strongly a country acts as a bridge with other countries in the ERA-LAC network. Centrality indices are essential tools for the analysis of networks as they are designed to rank the actors (e.g., countries) according to their position in the network, which can be viewed as the prominence of actors embedded in a network structure. Most centrality indices are based on shortest paths linking pairs of actors, measuring, e.g., the average distance from other actors, or the ratio of shortest paths an actor lies on. Following [17], publication networks can be described as a graph G = (V, E), where the set V of vertices represents countries of all authors in the publications, and the set E of edges represents links between countries. We can define a path from s ∈ V to t ∈ V as a sequence of vertices and edges, beginning with s and ending with t, such that each edge connects its preceding with its succeeding vertex. The length of a path is the sum of the weights of its edges. Let σ_st_ denote the number of shortest paths from s to t, where σ_ss_ = 1 by convention. Let σ_st_ (υ) denote the number of shortest paths from s to t that some υ ∈ V lies on. The betweeness centrality C_B_(υ) of each country can be calculated as (Eq 1):

$$C_{B}(\upsilon )=\sum_{s\neq \upsilon \neq t\in V}\frac{\sigma_{st}(\upsilon )}{\sigma_{st}}$$ (1)

#### Authorship, themes, ecosystems, and interdisciplinarity

We identified the country of the corresponding authors of all the ERA-LAC publications that study biodiversity and climate change together to see which countries were the main scientific leaders of these publications. To examine whether effects attributed to countries might actually be the work of a small number of prolific individuals, institutions or projects, we measured a diversity index on author, affiliation and funding fields of our database, using the probability of interspecific encounter (PIE, [18]). PIE expresses the probability that two publications randomly drawn from our survey correspond to different authors/institutions/projects:

$$PIE=(\frac{N}{N-1}){\left(1-\sum_{i=1}^{N}(\frac{N_{i}}{N}\right)}^{2})$$ (2)

where N = 500 (the total number of ERA-LAC publications in our database) and N_i_ is the number of individuals of the i^th^ author/institution/project in the database. For PIE < 0.70, the lower the PIE value, the more uneven its abundance distribution, i.e. it would be increasingly characterized by the dominance of one or a few authors/institutions/projects [18].

To assess how involved different scientific communities were in ERA-LAC collaborations on issues related to biodiversity and climate change, we also computed 1) the proportion of ERA-LAC publications at the interface of biodiversity and climate change that correspond to five major scientific disciplines defined by the WoS (biological sciences, earth sciences, technology, human sciences, medicine), 2) several themes in each discipline (for example, within earth sciences we identified the following themes: geography, geology, oceanography, atmospheric sciences, and water resources). Several themes could be attributed to a single publication (this occurred for about 35% of all publications). For publications classified as ‘biological science’, which made up most of the records, we also identified whether the study was performed in terrestrial, marine or freshwater ecosystems, and whether the study had been performed within an LAC country or within the ERA. Better-resolution ecosystem units (e.g. corals, coastal zone, pelagic zone, mountains, dry forest, rainforest etc.) were not included in our analysis as 1) many articles spanned several ecosystem units (e.g., survey of disease vectors, global monitoring of birds), 2) several articles concerned systems that were related to several ecosystem units (e.g., glacial streams and riparian vegetation, forest and ponds of the Pantanal), 3) many studies were conducted in very singular ecosystems occurring only a few times in the database (e.g., cloud forest, paramos, altiplano deserts). Therefore, any analysis on ecosystem specificities would have been poorly relevant. In addition, we used a Venn diagram to visualize the interdisciplinarity of the ERA-LAC research on biodiversity and climate change, distinguishing three large disciplinary fields: biological, earth and human sciences.

## Results

### Number of ERA-LAC publications on biodiversity and climate change, and temporal trends

**Fig 1:**
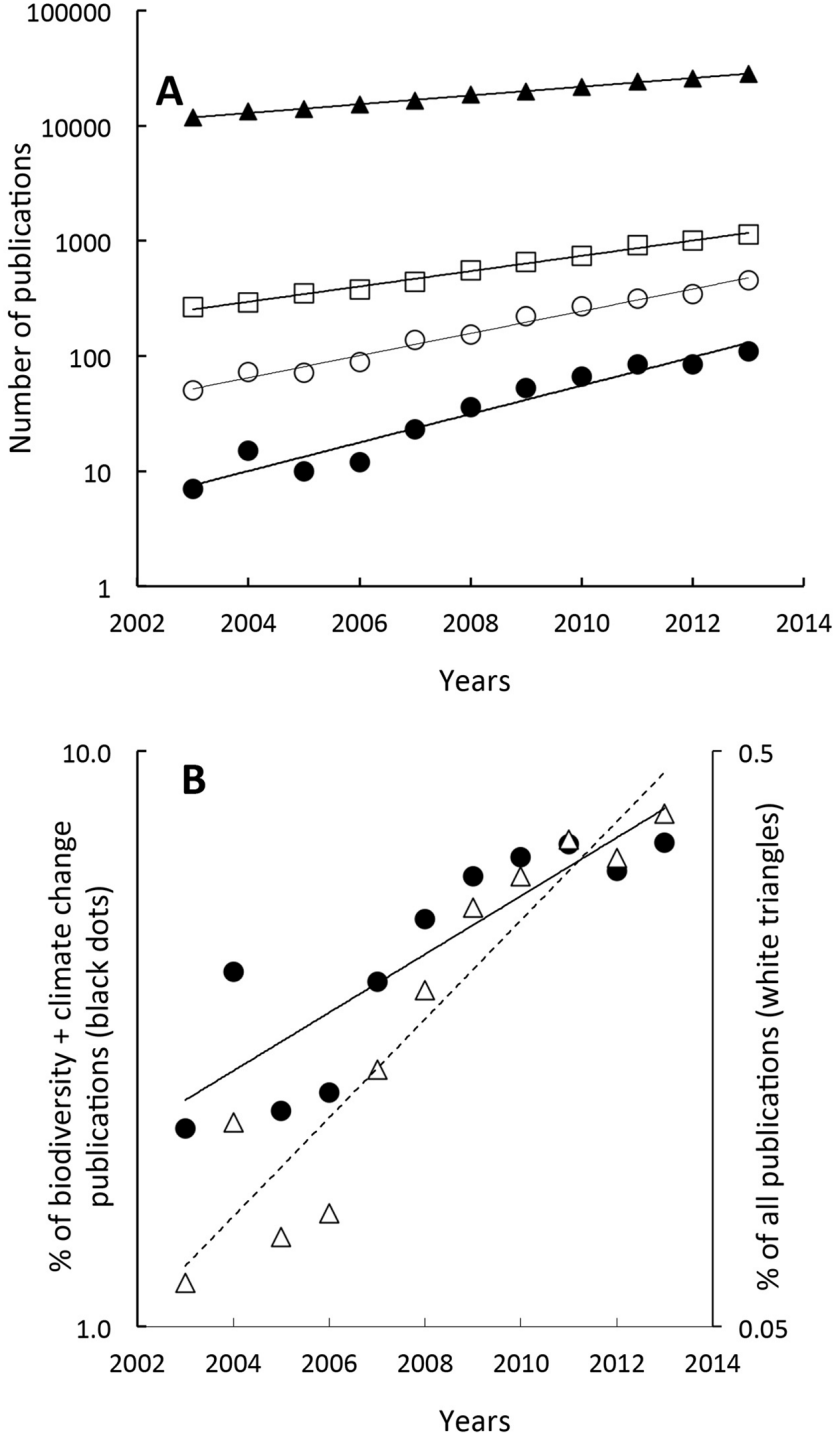
Temporal variation of ERA-LAC scientific publication thematics. (A) Temporal trend of the number of scientific publications (log scale) involving collaboration between Latin America/Caribbean (LAC) and the European Research Area (ERA) during the last decade: (White dots) climate change; (White squares) biodiversity; (Black dots) biodiversity and climate change; (Black triangles) all other topics. (B) Temporal evolution of the annual proportion of the total number of publications on biodiversity plus those on climate change (black dots, black line) and of the total number of publications on all other topics (white dots, dashed line) that were at the interface of biodiversity and climate change over the 10 year period studied.

The number of peer-reviewed publications that arose from collaborations between ERA and LAC countries on biodiversity, climate change and at the interface of biodiversity and climate change all increased exponentially between 2003 and 2013 (Fig 1A), with the highest exponential coefficients for the biodiversity/climate change interface (biodiversity/climate change = 0.28; climate change = 0.22; biodiversity = 0.15). As a control trend, the exponential coefficient for the publication number between ERA and LAC on all topics other than biodiversity and climate change was only of 0.087 during the same period. The total number of ERA-LAC co-publications at the interface of biodiversity and climate change between 2003 and 2013 was 500. The proportion of publications corresponding to this interface as compared to the sum of publications on ‘biodiversity’ plus ‘climate change’ increased 3-fold over the last decade from 2.2% to 6.9% (significant linear increase, R2 = 0.81, p < 0.01, Fig 1B). In parallel, a 8-fold increase was observed for the proportion of publications on the biodiversity/climate change interface regarding the sum of publications on all other topics (dash line, R2 = 0.92, p < 0.01, Fig 1B).

### ERA-LAC collaboration and its importance at the global scale

At a global scale, ERA-LAC co-publications represented 7.9% of the total number of publications at the interface of biodiversity and climate change between 2003 and 2013, co-publications between the ERA and North America representing 22.9%. Between 2003 and 2013, Europeans authored 2.8 times more publications at the interface of biodiversity and climate change than did LAC scientists (3706 vs. 1312, respectively).

**Fig 2:**
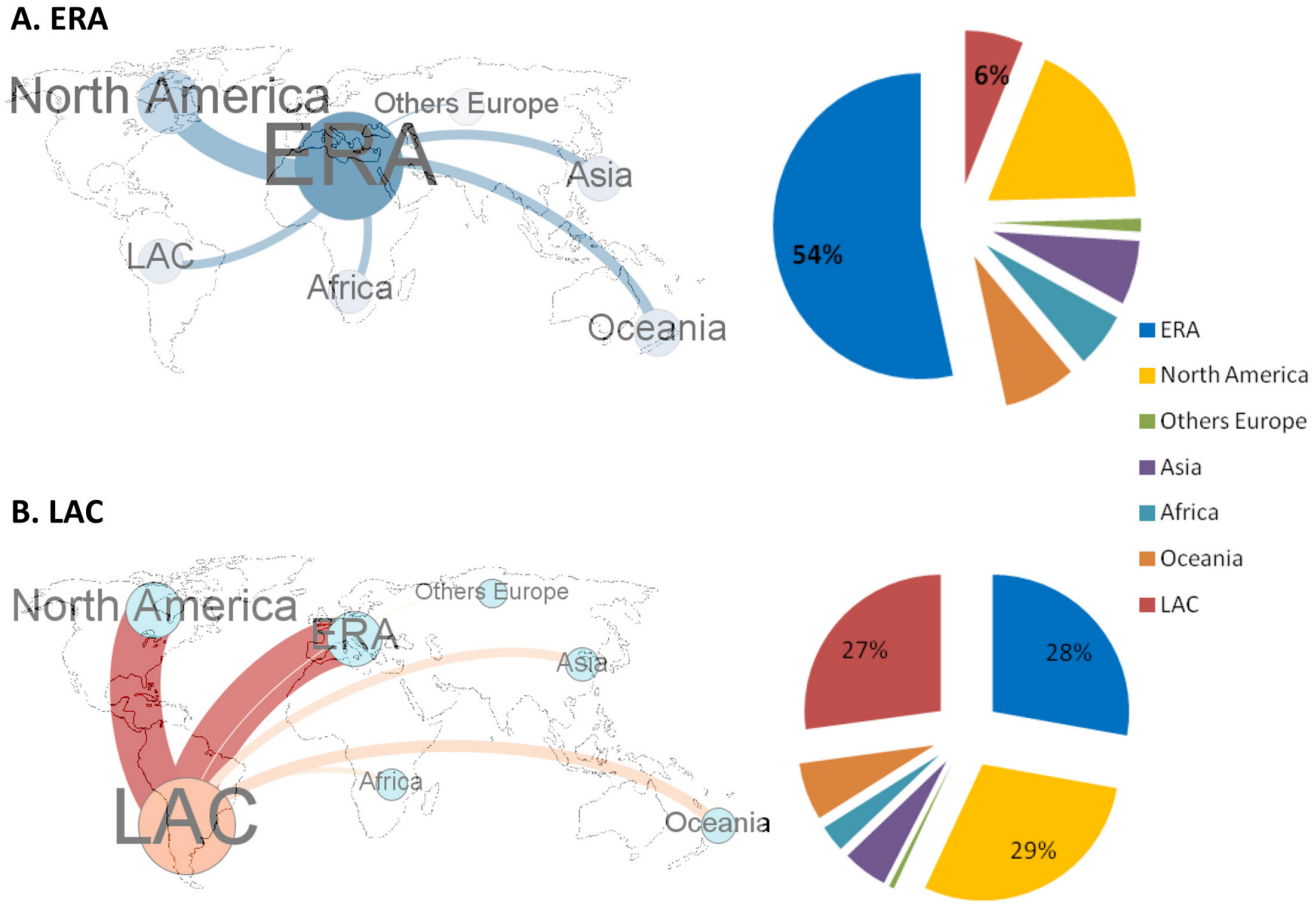
Strength of the research collaboration between LAC and the ERA. Data concern publications the interface of biodiversity and climate change as compared with other transcontinental collaborations on this topic for (A) the ERA and (B) LAC. The arrows in the maps represent the level of co-publication between two continents (node sizes are not comparable between the two panels). The pies on the right provide the values of the different links, including collaborations between countries within each region.

Europeans published studies at the interface of biodiversity and climate change mainly through intra-European networks (54.8% of all ERA publications on this topic), and to a lesser extent with North America (18.2%; Fig 2A). ERA-LAC collaborations (500 publications) were comparable to those between the ERA and Africa (467 publications), Asia (543 publications) and Oceania (631 publications).

**Fig 3:**
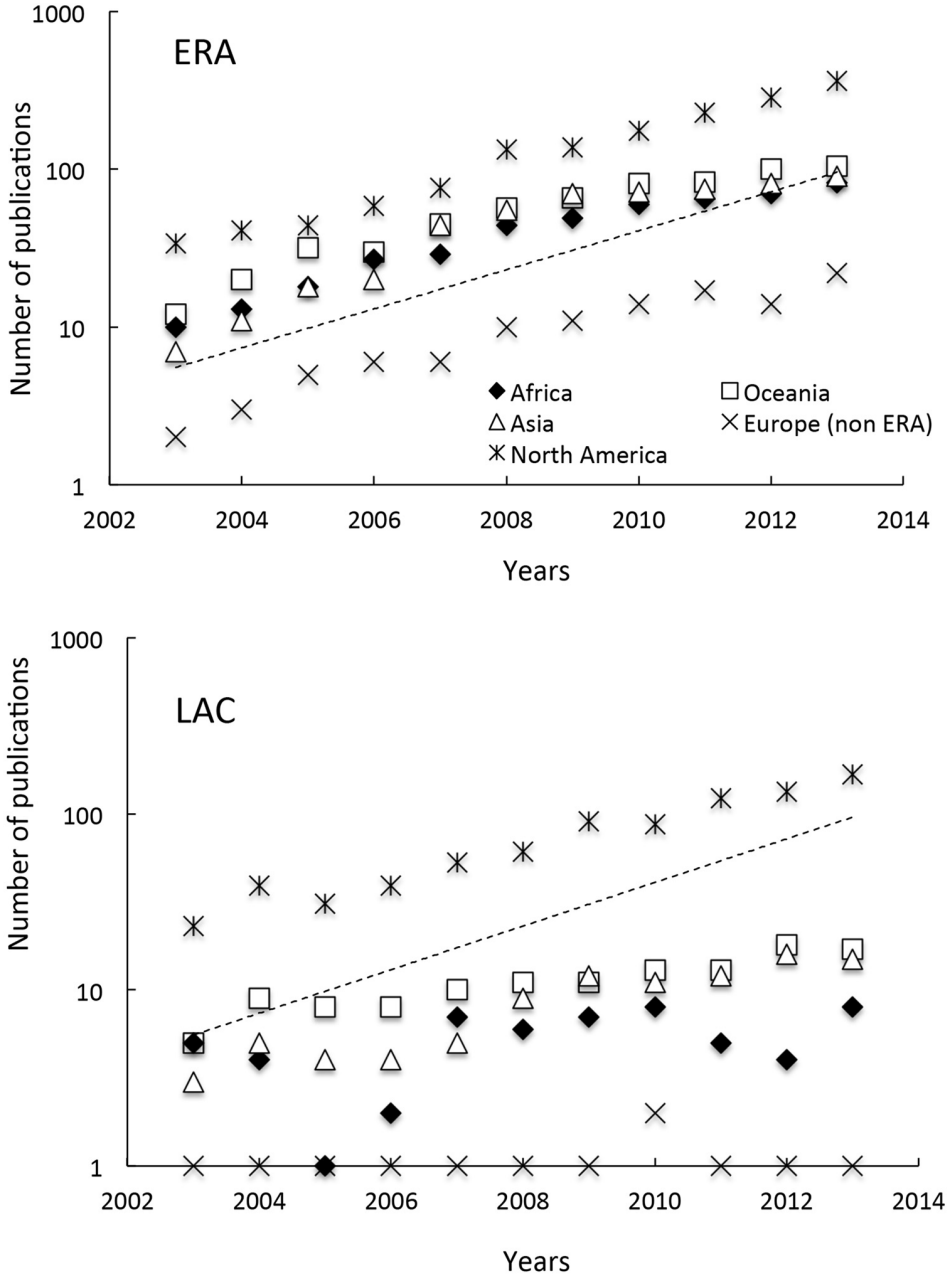
Temporal variation of ERA-LAC scientific publications with different continents. Temporal variation in the number of (Top) ERA and (Bottom) LAC publications on biodiversity X climate change that involved co-publications with either Africa, Oceania, Asia, Europe (non-ERA) or North America. The dashed line corresponds to the temporal trend observed for the ERA-LAC publications (see Fig 1A).

LAC scientists published studies at the interface of biodiversity and climate change equally with the ERA, North America and other LAC countries (27.0 to 29.2%), and they rarely published with colleagues from Oceania, Asia and Africa (Fig 2B). The number of publications at the interface of biodiversity and climate change that arose from collaborations between ERA countries and North America, Oceania, Africa, Asia and other European countries all increased between 2003 and 2013 (Fig 3A), but the exponential coefficients were lower (0.21–0.24) than those calculated for the ERA-LAC collaboration (0.28). Moreover, for LAC countries, the collaboration with Oceania, Africa, Asia and European countries other than ERA, only slightly increased over the 2003–2013 period (exponential coefficient range = 0.09–0.19).

### ERA-LAC collaboration network, and its temporal evolution

**Fig 4:**
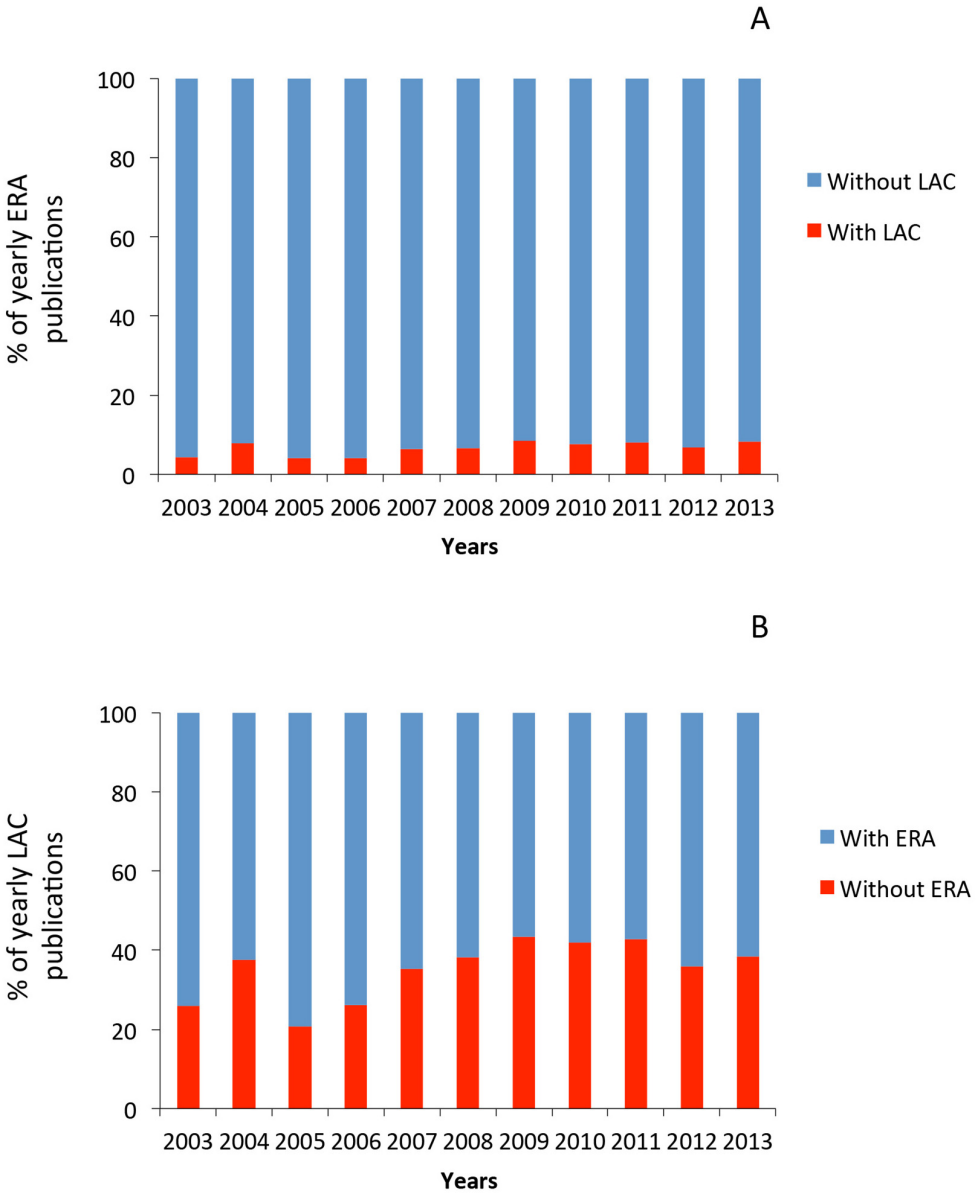
Temporal variation in the proportion of ERA-LAC publications. Temporal variation in the proportion of (A) the ERA publications that involve co-publications with LAC, and (B) the LAC publications that involve co-publications with ERA. The topic considered is the interface of biodiversity and climate change.

**Fig 5:**
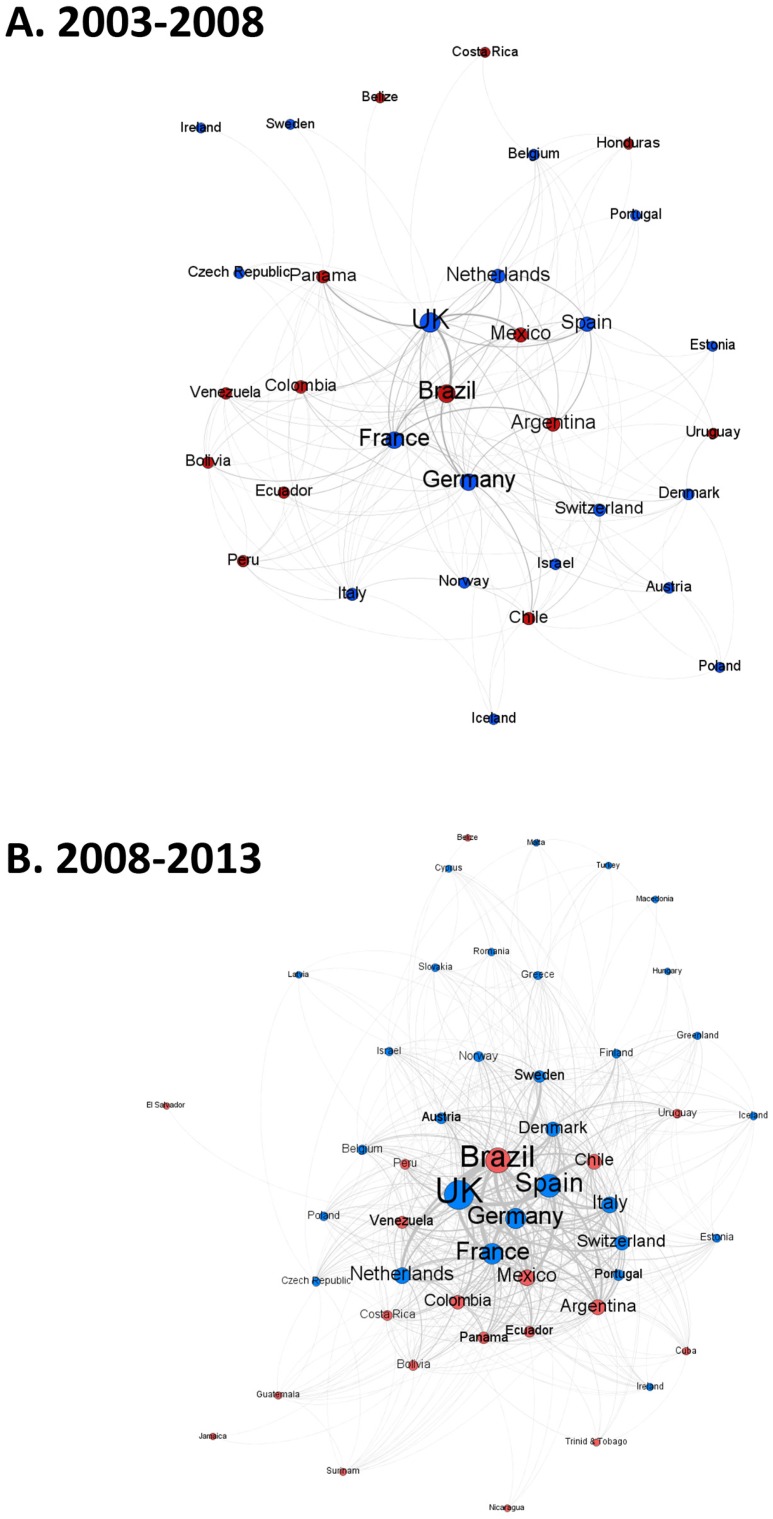
Cooperation networks of ERA-LAC publications. Cooperation network between countries from the ERA (in blue) and LAC (in red) on biodiversity and climate change together during (Top) 2003–2008 and (Bottom) 2008–2013. Disc size corresponds to the weighted degree (number of countries to which a country is linked, weighted by the number of publications).

The proportion of ERA-LAC co-publications at the interface of biodiversity and climate change increased from around 3% in 2003 to 8% in 2013 of total ERA publications (significant linear increase: R^2^ = 0.44, p < 0.05) and from around 25% to 40% of total LAC publications over the same period (Fig 4) (R^2^ = 0.41, p < 0.05). The increase in collaborative ERA-LAC papers at the interface of biodiversity and climate change (4.8 more papers in 2008–2013 than in 2003–2008) over time was associated with an expanded and more complex network of co-author links (Fig 5). On average, ERA and LAC countries increased their number of papers between the two time periods by a factor of 5.1 and 4.4, respectively. Brazil played an increasingly important role in the network, producing an average of 8.5 more papers between 2008 and 2013 than it did between 2003 and 2008. Several ERA countries increased their influence between the two periods as well: Sweden (30-fold increase), Portugal (25) and Italy (8.7). While the core of the collaboration network included Brazil, Argentina, Mexico, UK, Spain, France, Germany, the Netherlands and Switzerland for both periods, by the second time period that core also included Chile, Venezuela, Ecuador, Colombia, Portugal, Italy and Denmark (Fig 5).

**Fig 6:**
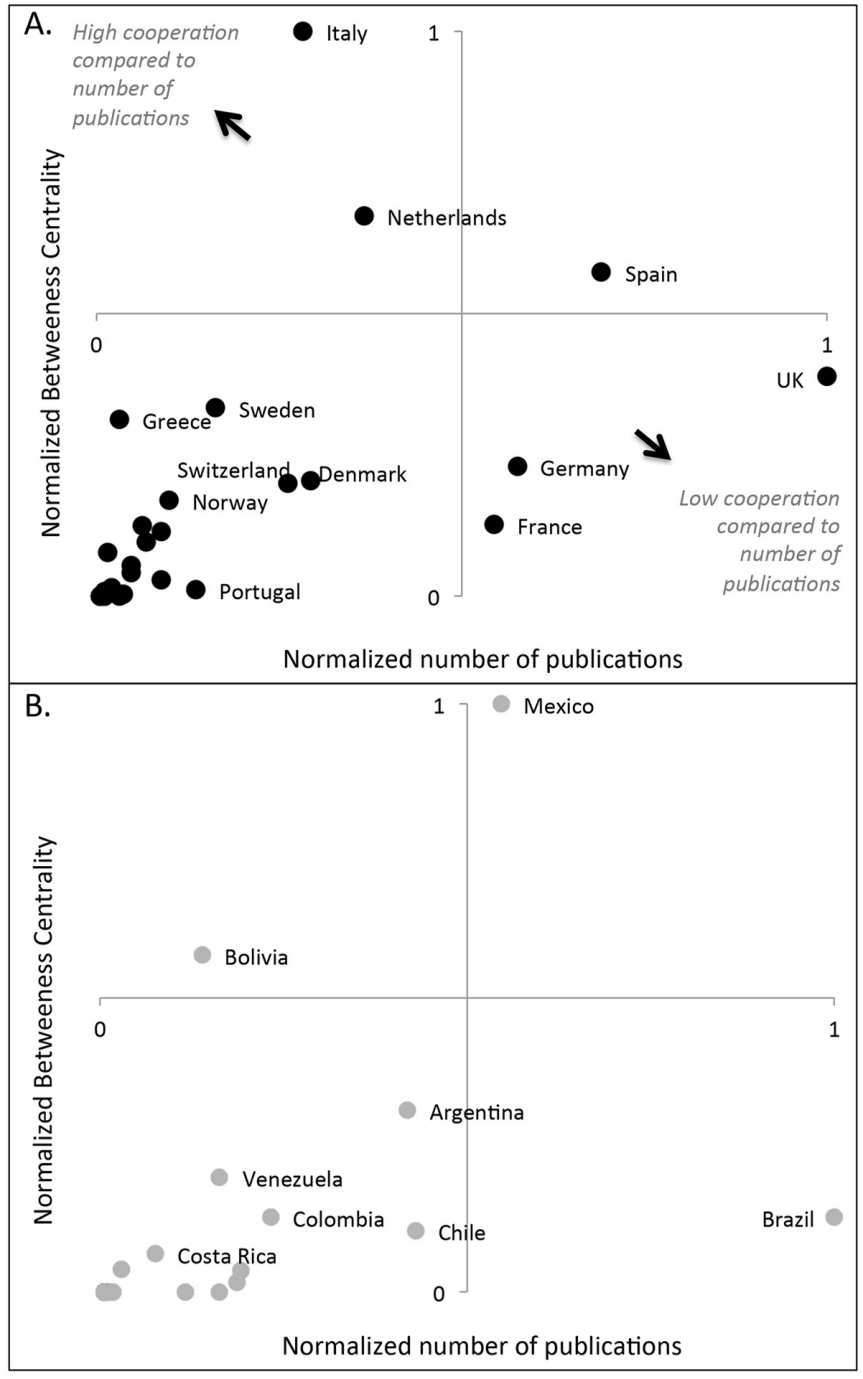
Betweenness centrality and number of publications involving cooperation at the interface of biodiversity and climate change between LAC and the ERA. (A) countries from the ERA and (B) countries from the LAC. For each variable and each panel, values are normalized by the maximum value observed.

Overall, a country’s influence in cooperative networks with the other countries (betweenness centrality) increased with the number of publications produced, i.e. countries with fewer publications generally had lower betweenness centrality coefficients (Fig 6). However, countries like, above all Italy and Mexico, but also Bolivia, the Netherlands and Spain had a higher betweenness centrality index than their publication number would suggest they should (Fig 6), while others, like the UK, Germany, France and Brazil (and to a lesser extent Chile) had a lower betweenness centrality score than might be expected based on their publication numbers.

### Leading authorship

**Fig 7:**
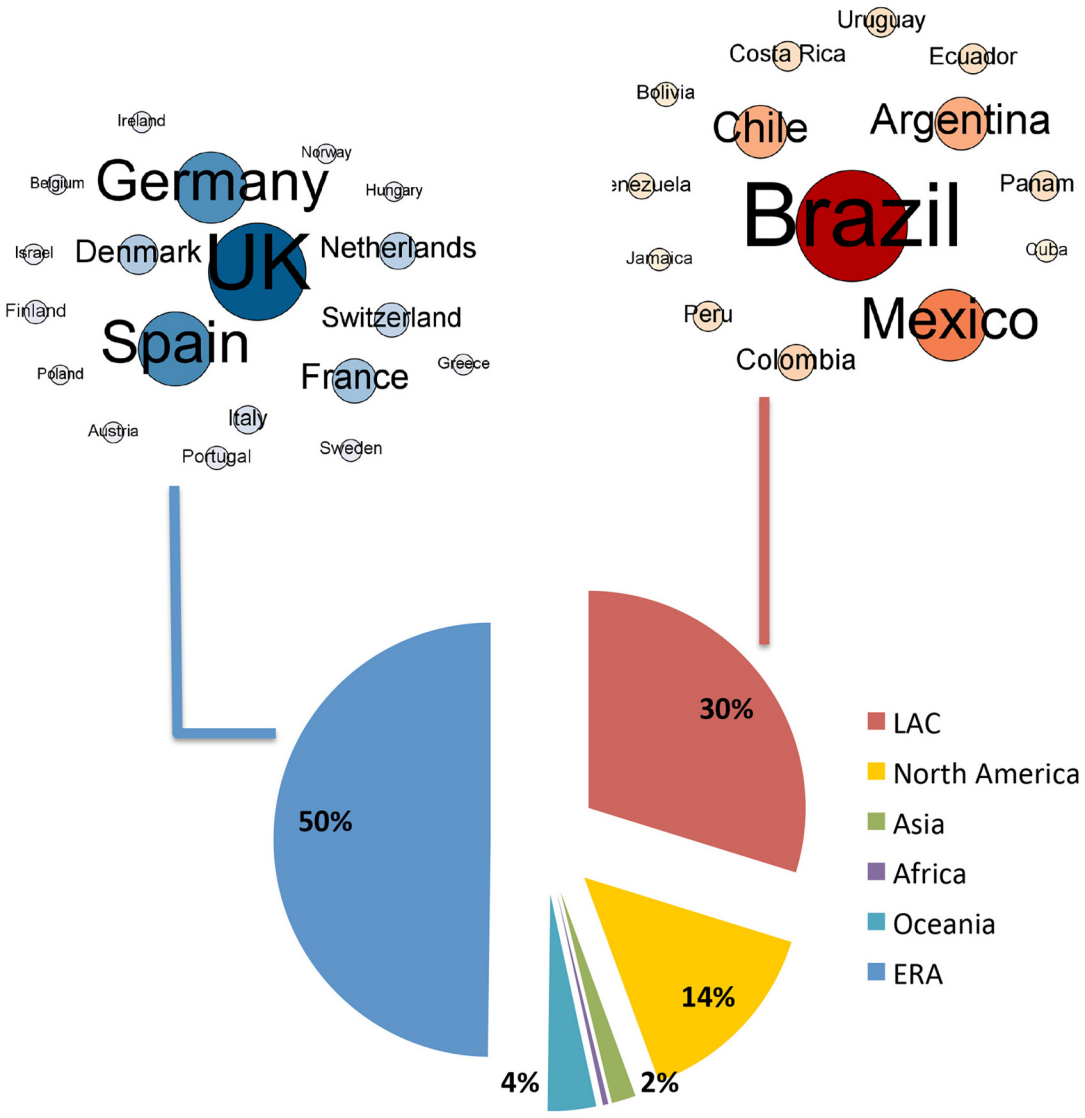
Geographic distribution of the corresponding authors of the ERA-LAC publications on biodiversity and climate change.

Overall, ERA researchers were corresponding authors for 50% of the ERA-LAC publications at the interface of biodiversity and climate change, while 30% were LAC researchers (Fig 7). ERA authors based in the UK, Germany and Spain were the most common for ERA-led ERA-LAC publications. LAC authors based in Brazil and Mexico were most common for LAC-led ERA-LAC publications (Fig 7).

No ERA-LAC authors, institutions or projects contributed disproportionally to the bulk of publication (PIE = 0.91, 0.87 and 0.89, respectively). The most prolific authors, institutions and projects accounted to a maximum of 2.0, 3.8 and 3.1% of all publications, respectively).

### Themes addressed, disciplines mobilized and interdisciplinarity level

**Fig 8:**
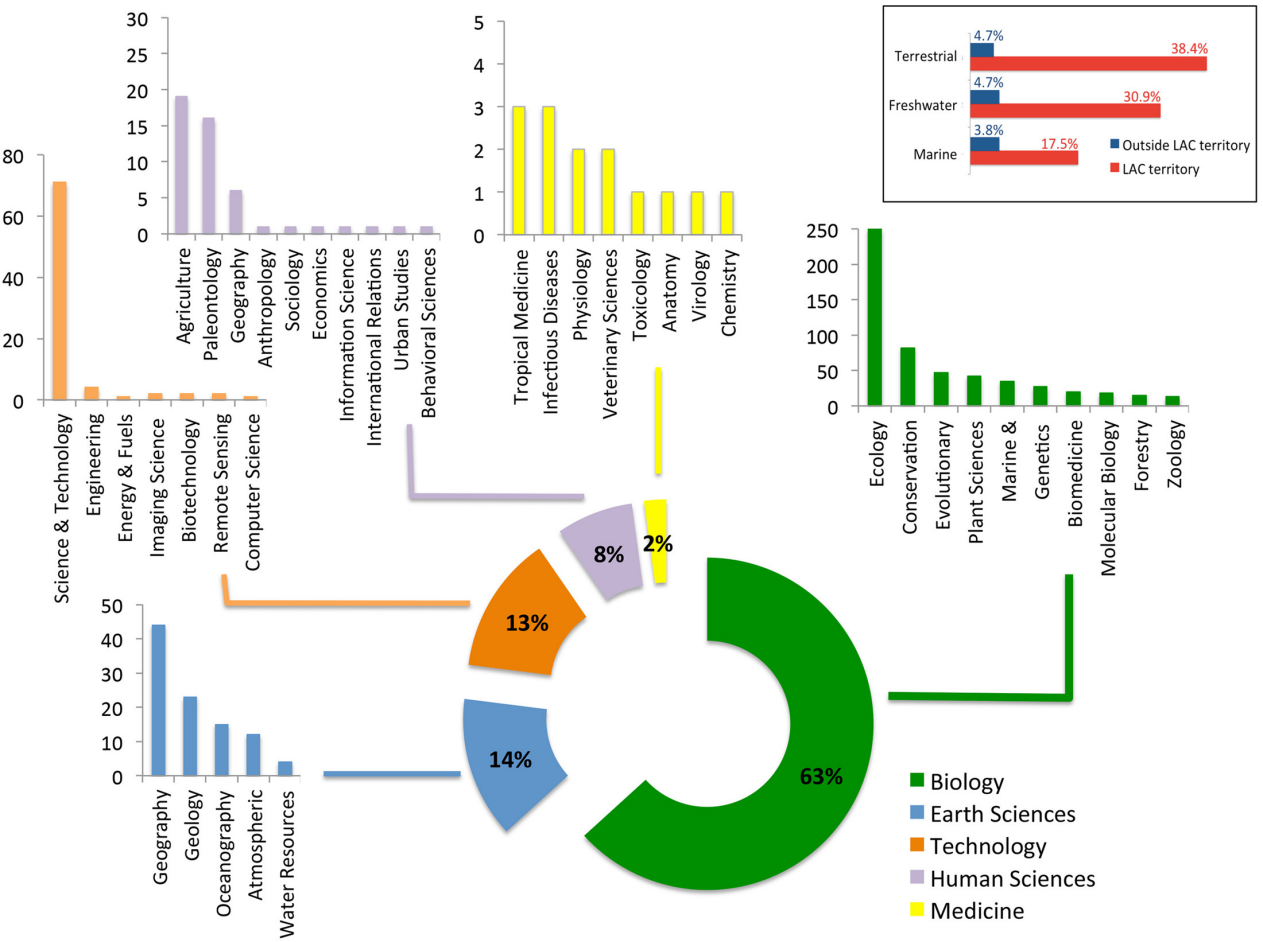
Major domains addressed by ERA-LAC co-publications on biodiversity and climate change. The inset (top right) details the environments studied, indicating whether or not the study sites were on LAC territory. Note that a given discipline (e.g., geography) can be classified into two different domains (e.g., earth sciences and human sciences)

Biological science was by far (63%) the most common theme among the ERA-LAC publications at the interface of biodiversity and climate change, followed by earth sciences (14%), technology sciences (13%) and human sciences (8%) (Fig 8). The most common scientific domains within these themes were ecology (250 publications), biodiversity conservation (82 publications), science and technology (72 publications) and physical geography (44 publications). Publications in domains of high socio-economic relevance, such as agriculture, fisheries or tropical diseases, were rare.

**Fig 9:**
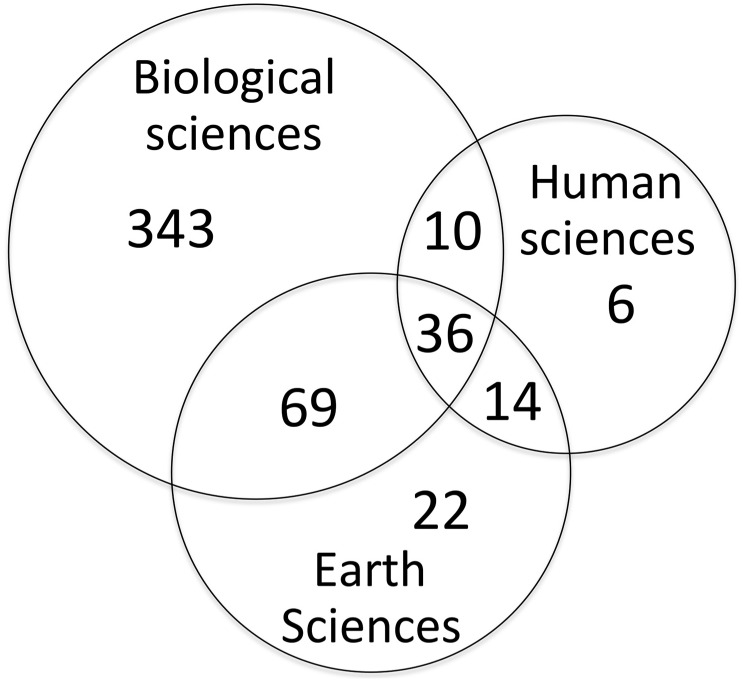
Venn diagram representing the level of transdisciplinarity among biological, earth and human sciences in the ERA-LAC co-publications at the interface of biodiversity and climate change. Circle diameter is proportional (log-scale) to the total number of articles.

Overall, 85% of all studies in biological sciences were conducted in sites/organisms within LAC territories, mainly in terrestrial and freshwater environments, and to a lesser extent, marine environments (Fig 8—inset). Interdisciplinary publications, at the interface of biodiversity and climate change, represented about one fourth (25.8%) of all publications in the ERA-LAC network (Fig 9). Publications that spanned both biological and earth sciences were most common (105 publications) followed by those arising from both human and earth science (50 publications), biological and human sciences (46 publications) and biological, human, and earth sciences (36 publications).

## Discussion

Within the LAC region is an exceptionally rich diversity of species and ecosystems. There is already severe pressure on the capacity of these species and ecosystems to deliver essential services to society [19, 20, 21]. While sound management of biological resources may strengthen the economies of the region, mismanagement in some countries is accelerating environmental degradation, food and water insecurity, and health and social problems [22]. The additional challenges imposed by climate change require the scientific community to join forces in documenting the climate change-induced alterations to ecosystems, increasing understanding of the biological underpinnings of ecosystem services (in particular, those linked to climate regulation, and climate change effects on services) and ecosystem-based adaptation [23], and developing other approaches for maintaining and restoring resilient socio-ecological systems in the face of climate change [24]. Our analysis shows that most ERA-LAC studies focus on sites/organisms within LAC territories, which demonstrates that ERA-LAC collaboration mainly aims at better studying issues related to biodiversity and climate change in this biodiversity-rich and climate-sensitive region.

Growth in the number of papers at the interface of biodiversity and climate change has been exponential over the last decade, indicating that scientists in the ERA and LAC have recognized the importance of studying the links between biodiversity and climate change. The proportion of publications at the interface of biodiversity and climate change and of publications involving ERA-LAC partners has also significantly increased between 2008 and 2013 than it did between 2003 and 2008. This increase in ERA-LAC publication in biodiversity and climate change was higher than that for other topics and with other regions of the world, even North America. In addition, although EU development policy aims to support Latin American regional integration [25], our study reveal strong differences in international collaboration patterns within LAC: there were much fewer co-publications with low- and lower-middle-income countries and much more with emerging countries like Mexico and Brazil.

These two main characteristics of the ERA-LAC collaborations at the interface of biodiversity and climate change, i.e. growth in collaborations and marked differences in representation among countries, have already been reported in other studies on international scientific cooperation, both between ERA-LAC regions [15] and worldwide [24]. Various theories have been proposed to explain the rise of international scientific cooperation [24]. Among them, the development of ‘big science’, including global biodiversity projects (e.g., GLORIA, CTFS), historical relationships like former colonial ties (e.g. within the Spanish-speaking communities), and development of national research funding programmes (e.g., in Brazil or Ecuador) have likely contributed to the expansion of the ERA-LAC co-publication network working at the interface of biodiversity and climate change. Moreover, it is noteworthy that the EU program for research and innovation has been opened to international cooperation under various schemes (bilateral, multilateral), thus encouraging international research projects to emerge, as in the case of ERA-LAC collaboration. Bilateral science funding and development policy are therefore also at play to explain the increase in ERA-LAC collaboration and associated number of papers in the study period. These contribute to integrating research cooperation and policy into development cooperation processes, although it has to be differentiated from the official EU international cooperation and development programs (e.g., EuropeAid), which has less to do with scientific research policy.

The increase in scientific co-authorships between ERA-LAC countries is also likely an outcome of the increasingly complex web of relationships that has been weaved between scientists of the two regions [15]. While some countries like Mexico, Italy and the Netherlands relied heavily on international and regional collaboration to boost scientific production, others like Brazil and the UK appear to have another strategy by engaging in bilateral collaboration with selected countries or states [15]. Overall, countries like Brazil, France, the UK and Germany mostly develop bilateral cooperation with poor regional integration, and further efforts should be made to foster cooperation within LAC if regional integration is viewed as a key target. While no individual ERA country reached levels of co-authorship like that between the United States of America and LAC, the ERA as a whole equalled North America’s level of scientific partnerships with LAC. This pattern probably illustrates comparable economic units and funding flows between North America/ERA and LAC [7].

Many issues addressed by the scientific community studying biodiversity and climate change together (e.g., the study of reef system’s resilience to climate change or adaption to changing fishery resources) requires an interdisciplinary approach that includes life, earth and social sciences. Our study found that about one fourth of all studies on biodiversity and climate change within the ERA-LAC cooperation network were interdisciplinary. These levels may be underestimated, as our publication sample was not selected on terms that might have pulled in more human science papers. Moreover, within the large categories are also many disciplines and important interdisciplinarity which may be overlooked. While our study cannot conclude on the importance of interdisciplinarity in the ERA-LAC network, this issue is central to sound research on biodiversity and climate change and would merit further investigations. As evidenced in a recent ALCUE-Net meeting (see http://alcuenet.eu/meeting-events.php?event=TkRRNA), a major barrier to the scientific cooperation between biodiversity and climate change communities seems to be the discrepancy in the data formats, resolution, uncertainties, and access of each community. To improve understanding and collaboration between both communities, their dialogue should be strengthened through capacity building activities and cooperation projects so that biodiversity community’s expectations could be better identified and climate models limitations better assessed. Moreover, although the internal disciplinary differentiation of science motivates linkages among research teams in specific research domains at the international level [26], the scientific community of both regions must put more effort into developing common study models and systems. This would help integrate a greater breadth of expertise, foster participatory science, and ultimately provide knowledge to decision makers consistently with the frameworks of the Intergovernmental Panel on Climate Change (IPCC) and IPBES.

Over the last twenty years, the ERA and LAC countries have committed themselves to consolidating their links through a strategic partnership. A major objective of these reinforced links is often to build countries’ capacity. However our analysis shows that while most ERA-LAC studies are performed on sites located in LAC territories only one third of the corresponding publications are led by LAC scientists. However, the relationship between capacity building and publication authorship is not straightforward and the poor leadership of LAC scientists may also illustrate the ERA origins of the funding and lack of research coordination in the LAC region. A second major objective is to promote academic excellence and productivity. Further parameters like journal impact factors and corresponding author metrics could be incorporated into bibliometric analyses to assess whether ERA-LAC collaboration has developed in a way that fulfils this goal. A third major objective of reinforced ERA-LAC links is to properly study the interplay of biodiversity and climate change issues over the entire LAC area. However, our results indicate that ERA-LAC collaboration is biased towards a few, emerging countries, with most studies focused on sites in Brazil and Mexico (which is a powerful actor of intra-LAC cooperation), and there is a need to better tailor cooperation with low- and mid-income countries by conducting more targeted collaborations for R&D studies on biodiversity and climate change. However, strengthening the connection between less developed countries and the global cooperation network will not be easy. For example, Bolivia appears in our analysis only as a peripheral link to the global network through regional hubs, but it has very limited academic resources in biodiversity and climate change science. The limitations of climate model downscaling (for example in the Andean region, [13]) and the fragmented information available on the conservation status of many South American species [27] illustrate the urgent need for ERA-LAC initiatives at the interface of climate change and biodiversity to integrate a truly regional approach. As it is unlikely that such an initiative would come from individual countries, the EU faces a key challenge in promoting regional scientific cooperation in LAC countries. It is a daunting task, but the recent calls launched by ERA-LAC networking projects such as the ERANet-LAC (http://eranet-lac.eu, €6 millions in 2015–2016) are promising advances to address this issue.

## Supporting Information

S1 Table Complete database of publications used for the analysis. (TXT)
